# Omics Approaches to Identify Potential Biomarkers of Inflammatory Diseases in the Focal Adhesion Complex

**DOI:** 10.1016/j.gpb.2016.12.003

**Published:** 2017-04-01

**Authors:** Johanne Brooks, Alastair Watson, Tamas Korcsmaros

**Affiliations:** 1 Gut Health and Food Safety Institute Strategic Programme, Institute of Food Research, Norwich Research Park, Norwich NR4 7UA, United Kingdom; 2 Norwich Medical School, University of East Anglia, Norwich Research Park, Norwich NR4 7TJ, United Kingdom; 3 Gastroenterology Department, Norfolk and Norwich University Hospital, Norwich NR4 7UY, United Kingdom; 4 Earlham Institute, Norwich Research Park, Norwich NR4 7UZ, United Kingdom

**Keywords:** Focal adhesion complex, Biomarkers, Inflammation, Omics, Systems biology

## Abstract

Inflammatory diseases such as inflammatory bowel disease (IBD) require recurrent invasive tests, including blood tests, radiology, and endoscopic evaluation both to diagnose and assess disease activity, and to determine optimal therapeutic strategies. Simple ‘bedside’ **biomarkers** could be used in all phases of patient management to avoid unnecessary investigation and guide further management. The **focal adhesion complex** (FAC) has been implicated in the pathogenesis of multiple inflammatory diseases, including IBD, rheumatoid arthritis, and multiple sclerosis. Utilizing **omics** technologies has proven to be an efficient approach to identify **biomarkers** from within the FAC in the field of cancer medicine. Predictive **biomarkers** are paving the way for the success of precision medicine for cancer patients, but inflammatory diseases have lagged behind in this respect. This review explores the current status of biomarker prediction for inflammatory diseases from within the FAC using **omics** technologies and highlights the benefits of future potential biomarker identification approaches.

## Introduction

**Figure 1:**
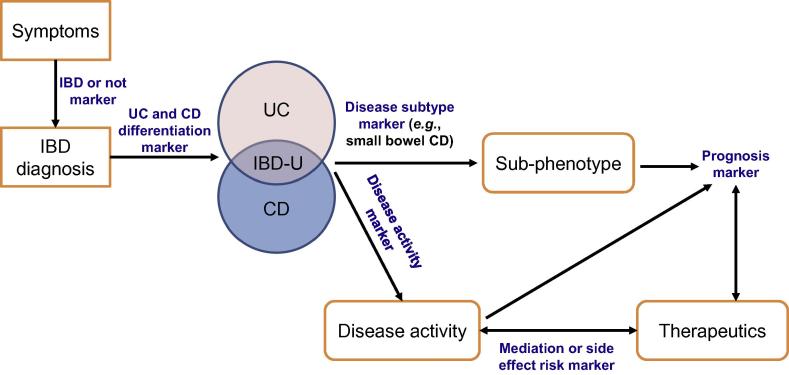
**Potential sites of biomarker used in IBD** BD-U refers to IBD-undefined, for which the patient’s endoscopy and histology cannot give a clear distinction between UC and CD. IBD, inflammatory bowel disease; UC, ulcerative colitis; CD, Crohn’s disease.

Disease biomarkers have the potential to be medically valuable at all stages of the disease process from diagnosis, identification of disease subtypes, and prognosis to therapeutic adjustment. Inflammatory bowel disease (IBD) is an exemplar of a chronic, complex inflammatory disease. IBD has two major subtypes, ulcerative colitis (UC) and Crohn’s disease, which have different clinical courses and management strategies with a wide phenotypic variability among patients. Figure 1 highlights the points at which biomarkers have potential use in IBD.

**Figure 2:**
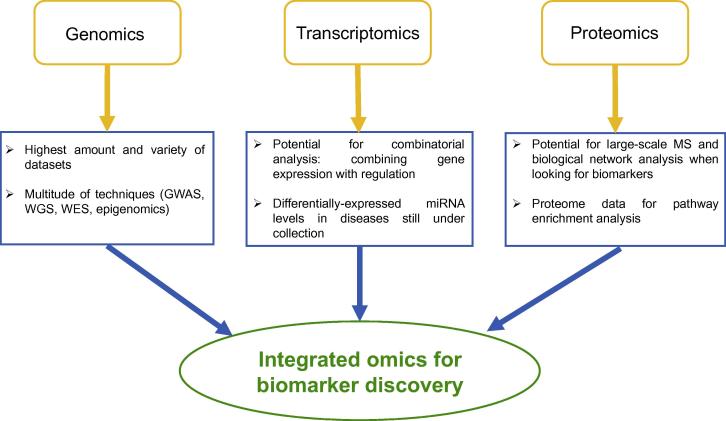
**Omics approaches with complementary potential to be integrated** Genomics, transcriptomics, and proteomics approaches can be used to identify and discover the detailed component, mechanisms, and regulation of the FAC members in normal and in diseased states. The differential analysis is capable to point out novel biomarkers. FAC, focal adhesion complex; GWAS, genome-wide association studies; MS, mass spectrometry; WES, whole-exome sequencing; WGS, whole-genome sequencing.

Biomarkers need to be specific, stable, and consistent across multiple platforms of testing in order to be used as a clinical application. This raises challenges associated with biomarker identification in IBD, as with any complex inflammatory condition, partly due to our limited understanding of the pathogenesis of these diseases and poor appreciation of the difference between what is healthy and what is a disease process. Hypothesis-driven biomarker discovery via traditional one protein–one metabolite or one cell analysis from cellular disease models or tissues compared between control and disease samples is laborious. Such an approach is also limited by the fact that gene expression and signalling of tissues depends on the context and their native environments [1]. For this reason, very few biomarkers make it to clinical practice [2]. Further challenges posed by complex diseases are that they often need to be stratified into sub-phenotypes via patients’ genetic features, which need to be taken into account, making identification of a broad generalizable biomarker difficult [3]. High throughput, hypothesis-free techniques are required for biomarker discovery. With the advent of high-throughput omics technologies and advances in computational biology, researchers are now able to generate, analyse, and interpret a variety of datasets and apply them on biomarker discovery at a scale, which were previously impossible (Figure 2). One of the cellular signal transduction pathways supplying candidate biomarkers that have become prominent through the use of omics technologies and computational biology, certainly for the cancer field, is the focal adhesion complex (FAC).

FACs are dynamic, large protein assemblies that mechanically link and transduce signals from the extracellular matrix to the intracellular milieu via integrins [4] or other receptor modules such as cluster of differentiation 47 (CD47). The complex consists of core structural proteins such as paxillin, talin, actinin, and vinculin, with dynamic signalling proteins including protein kinases, phosphatases, small guanosine triphosphatases (GTPases) with regulatory molecules, and adapter molecules that mediate core protein–protein interactions (Table 1). The ‘adhesome’ network contains 156 components with 690 interactions between them [26], highlighting the complexity of the focal adhesion function.

The focal adhesion function is both mechanical and responsive. It is mechanical in terms of anchoring the cell to the extracellular matrix via binding of integrins to their extracellular ligands and to the actin cytoskeleton to modify the physical and topographical characteristics of the cell. This has direct implications for wound healing as well as invasion and the metastatic nature of the cancer cell. The responsive function of the FAC is diverse and multi-layered. Depending on the initiating signal, FAC can be involved in regulating inflammatory gene expression via signal transduction pathways such as interleukin 1 (IL-1) signalling [27], [28] or regulating calcium fluxes via phosphatidyl inositol signalling [29], which impact on inflammatory cascades. Many molecules in the FAC are involved in downstream signalling pathways, for instance, the MAPK/ERK pathway [30], AKT1 [22], and Wnt signalling [31], [32]. In this way, pathways impacted by the FAC are as varied as apoptosis [21], production of cellular protrusions [33], cell cycle progression [34], and cell proliferation [35].

**Figure 3:**
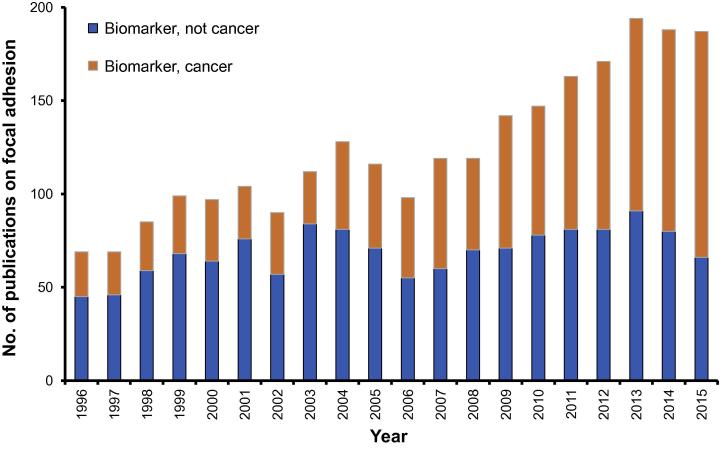
**Biomarker related publications about focal adhesion complex** We compared the total publications in PubMed identified with MeSH terms ‘focal adhesion and biomarker’ with ‘cancer, focal adhesion and biomarker’ from the last 20 years. The figure highlights the unchanged and low number of biomarker-related studies involving the FAC and non-cancer diseases compared to cancer related studies. FAC, focal adhesion complex.

The number of publications listed in PubMed involving FAC (‘focal adhesion complex’) has had a 5-fold increase from 141 published in 1996 to 709 published in 2015. The role of FAC in cancer has been a consistent focus of approximately 44% of publications over the past 20 years (Figure 3). Given the critical roles that focal adhesions play in regulating cell structure, proliferation, survival, migration, and invasion, it is not surprising that this makes the complex a prime target for biomarker candidacy and drug targeting in cancer, which is reflected in the overrepresentation of papers with the terms ‘cancer’, ‘focal adhesion’, and ‘biomarker’ from a cohort of ‘focal adhesion’ and ‘biomarker’ publication subset.

Of the publications identified using the Medical Subject Headings (MeSH) terms ‘cancer’, ‘focal adhesion’, and also adding ‘biomarker’, 39 out of 745 used bioinformatics approaches for biomarker identification. It is of note that all these 39 studies were published after 2007.

The role of FAC in the pathobiology of inflammatory diseases such as IBD or rheumatoid arthritis (RA) has been less well exploited for biomarker discovery. However, the role of FAC in inflammatory diseases can be well illustrated in UC. UC is a relapsing-remitting disease which causes ulceration of the lining of the large bowel and is thought to be a disease of the epithelial barrier [36]. The epithelial barrier is an immuno-mechanical barrier consisting of mucous layers, intestinal epithelial cells, and closely-residing immune cell populations. The mechanical barrier is provided in part by the enterocytes joined by intercellular junctions, of which the tight junction is a major component. May et al. [37] identified that activation of focal adhesion kinase (FAK) is necessary for maintaining and repairing the epithelial barrier in cell culture via tight junctions. This was further examined by Khan et al. [38] in both T84 cell lines and surgical specimens from IBD patients. They demonstrated that activation of M1 muscarinic acetylcholine receptor augmented the recovery of epithelial barrier function via phosphorylation of FAK. Further evidence for the role of FAK in maintaining intestinal epithelial barrier function in the presence of pathogenic factors was highlighted by Guo and colleagues [39]. Utilizing intestinal epithelial cell cultures, they identified that gut-derived bacterial lipopolysaccharide induced tight junction permeability via the FAK/myeloid differentiation primary response gene 88 (MyD88)/IL1 receptor pathway. GTPases such as Rac1 [40] and tyrosine phosphatase members of FAC have a role in regulation of the NACHT, LRR and PYD domains-containing protein 3 (NLRP3; also known as cryopyrin) inflammasome [41], which mediates the release of IL-1 and IL-18 from cells. IL-18 signalling drives the breakdown of barrier integrity in murine models of UC [42]. Further evidence of FAC involvement in inflammasome activation was provided by Thinwa et al. [43] who demonstrated that the initial signal for intestinal cell inflammasome activation in pathogen recognition is via integrins. It is interesting to note that *NLRP3* was identified as a candidate gene for susceptibility of Crohn’s disease [44], whereas IL-1 has been put forward as a faecal marker of inflammation in UC [45].

The evidence described above has been hypothesis-driven, utilizing mainly cellular models to describe a pathogenic system. In this review we will consider the literature field of FAC in inflammatory diseases focusing on those utilizing a systems medicine approach, where omics data and computational biology are combined for potential biomarker identification.

In the last two decades, omics technologies have made a great impact on medical research, turning biological research into a data-intensive science [46]. These high-throughput methodologies are now routinely used to provide a top-down approach in understanding biological systems. The power of omics approaches in systems medicine is due to their ability to detect context (*e.g.*, cell, disease, or treatment) specific data for a signalling system. The challenge of these approaches is that it often requires either a computational biology expert or familiarity with sophisticated computational software solutions to extract biological insights from the datasets [47]. A further complication is that genomic or transcriptomic data are often best interpreted in the context of the heterogeneous large-scale datasets that have already been deposited in publicly-available databases [47].

## Genomics

Genomic approaches provide the highest number and variety of datasets on human diseases. These approaches include (1) whole-genome or whole-exome sequencing that identify genetic mutations or copy number variations; (2) genome-wide association studies (GWAS) used to identify genetic variants associated with a disease; (3) microarray or RNA-seq techniques for measuring the mRNA or microRNA (miRNA) expression of cells and comparing the levels between states (transcriptomics); and (4) epigenomics analyses focusing on, for example, DNA methylation and its change during differentiation, ageing, and cancer progression. To analyse the genomic datasets of complex diseases, the systems medicine approach is a highly-effective framework to understand the complexity. Disease-related genes may differ among affected individuals, but the affected pathway or network region is likely to be shared [47]. The identified disease-related genes can be used to list potential biomarkers by filtering those specifically relevant to a given disease or disease stage.

In particular, the advent of GWAS identifying candidate susceptibility genes has opened the door to the pathobiology of chronic inflammatory disease. With this, the prospect of a genetic marker for disease diagnosis, prognosis, and therapeutic efficacy in what can otherwise be very heterogeneous diseases is very appealing. GWAS in large populations of patients with chronic inflammatory diseases such as RA can identify common genetic variants that are associated with having that disease [48].

Zhang et al. [49] undertook analysis of the KEGG pathways [50] affected by 11,922 differentially-expressed genes (DEGs), which had been identified by genome-wide association scans in RA patients. The focal adhesion and extracellular matrix receptor interaction pathways were considered high risk RA pathways. Core members of FAC with genetic variants included integrin subunits A and B, actinin, dedicator of cytokinesis 1 (DOCK1), and B cell lymphoma 2 (BCL2). Their data correlate well with the DNA methylome signature in RA, comprising genome-wide DNA methylation loci from fibroblast-like synoviocytes removed at the time of joint replacement from five patients with osteoarthritis and six patients with RA [49]. Nakano et al. [51] undertook global methylation status analysis and identified differential methylation between osteoarthritis and RA in 1206 different genes. Differentially-methylated genes were mapped to KEGG pathways for gene ontology, which highlighted hypomethylation enrichment in the RA sample in loci including genes encoding integrin subunits A and B, actinin, receptor tyrosine kinases, parvin, DOCK1, and BCL2. Hypomethylation of inflammatory genes has been associated with an increased inflammatory response, as hypomethylation in promoter regions of a gene makes it transcriptionally active [52], [53].

Utilizing GWAS-mapped genes or methylome signatures alone for biomarker prediction has its limitations. Firstly, the differential expression of said genes is not assessed. Secondly, the presence or absence of a single polymorphism within a gene may not have a strong enough phenotype to be a useful biomarker [54]. Moreover, the use of methylation status as a biomarker is currently plagued by inaccuracy and poor replication, as there is a need for standardized methods and controls [55].

To overcome the potential limitation of not taking into account differential gene expression, He et al. [56] examined the Gene Expression Omnibus (GEO) microarray data to assess mRNA expression in the specific cell type involved in RA, synovial fibroblasts, to identify DEGs by comparing six RA patients to osteoarthritis patients (an age related, non-autoimmune arthritis) using the linear models for microarray analysis (LIMMA) [57]. The authors undertook functional enrichment of the DEGs using KEGG pathways, with the analysis performed using the database annotation visualization and integrated discovery (DAVID) [58]. Using STRING [59], they created a larger protein–protein interaction (PPI) network for a further functional enrichment, looking for functional complexes using the MCODE plugin for Cytoscape [60]. This multi-layered approach comparing the two types of arthritis identified DEGs for collagen (a predominant member of the extracellular matrix) that were enriched in focal adhesion pathways and extracellular matrix receptor interactions for osteoarthritis, but not RA. The difficulty of biomarker identification based on gene expression studies only is that the studies are often small, thereby not taking into account the rich genetic variability of these complex diseases, and neither gene regulation nor protein levels of DEGs.

## Transcriptomics

Combinatorial approaches utilizing DEGs and their regulation have been more successful for biomarker discovery. One mechanism of gene regulation is via small non-coding RNAs (ncRNAs) such as miRNAs. miRNAs function in RNA silencing, by base pairing binding of complementary sequences in mRNAs, thus targeting them for cleavage [61]. In the field of oncology, integrating miRNA, gene expression, and transcription factor signatures has been used to identify biomarkers for papillary thyroid cancer by using pathway enrichment to identify dysregulated pathways including in focal adhesion [62]. Such approach of integrating miRNA data and differential gene expression for identification of molecular prognostic biomarkers was taken further by Cai and colleagues [63], who identified three potential biomarkers, *CALM2*, miR-19b, and miR181b, for gastric cancer that were related to the FAC and the extracellular matrix receptor. This integrative approach has been, however, less widely used in inflammatory models. For IBD [64] and many other autoimmune diseases including Sjogren’s disease [65], we are still at the stage of documenting differential expression levels of miRNAs between disease and control cohorts.

Therefore, despite the central role FAC plays in inflammatory diseases, the number of ncRNAs that could be used as potential biomarkers are still scarce. In the case of UC and Crohn’s disease, miRNAs are the most explored ncRNAs in the literature. There is experimental evidence showing elevated levels of specific miRNAs in active UC tissues and in serum [66].

In recent years, many computational methods emerged that allow the analysis of specific ncRNA–disease associations, predict such connections and select the ones most suitable for experimental validation. For example, heterogeneous graph inference for miRNA–disease association prediction (HGIMDA) [67] and improved random walk with restart for lncRNA-disease association prediction (IRWRLDA) [68] are two viable, novel methods that could be potentially used to describe new targets. HGIMDA constructs a heterogeneous graph out of separate networks: a functional similarity network of miRNAs and a semantic similarity network of diseases, which in combination allowed predicting potential disease–miRNA associations. IRWRLDA uses an improved random walk with restart algorithm on a lncRNA similarity network to rank potentially useful candidate lncRNAs.

## Proteomics

Protein biomarker identification is driven by better understanding of the disease processes and signalling pathways involved in perpetuation of pathogenic states. Combining large-scale mass spectrometry (MS)-based proteomics and biological network analysis has been fundamental in the understanding of signalling networks [69], so it stands to reason that using similar techniques may drive biomarker identification for the large datasets that have been proved by proteomic platforms. Like genomics and transcriptomics, biomarker discovery using proteomics has often involved proteome analysis with pathway enrichment. A good example of this is reported by Rukmangadachar and colleagues [70]. They differentiated intestinal tuberculosis (TB) and ileal Crohn’s disease, utilizing MS-based proteome analysis on ileal biopsies of 15 patients, in combination with pathway enrichment using KEGG pathways and the PANTHER annotation resource, and identified biomarkers of both intestinal TB and Crohn’s disease. They were able to identify overexpressed proteins in Crohn’s disease patients compared to intestinal TB patients. These proteins were annotated to pathways such as the integrin signalling pathway, including a core FAC member, vinculin. However, the proteins they identified were unable to be validated as differential biomarkers in their 52-patient validation cohort using immunohistochemistry. This emphasizes the point that a one-step, single-omics approach on a small cohort of patients, whilst identifying potential pathways, lacks the finesse to complete the biomarker discovery.

## Systems biology and focal adhesion — the promise for novel biomarker discovery

Looking again at the cancer model, we can see that integrative approaches using both omics data and computational biology have been successful in producing panel biomarkers for cancer subtypes. A good example of this is reported by Zhang and colleagues [71]. They took a systems biology approach to discover, characterize, and validate a panel of breast cancer biomarkers from breast cancer proteomics data. Using liquid chromatography (LC)-coupled MS data from 40 women with breast cancer and 40 women without breast cancer, they identified statistically significant differentially-expressed proteins. They further identified PPI networks and performed pathway analysis with significant literature curation (hypothesis-driven). As a result, they identified a panel of 25 breast cancer biomarkers, which were able to be validated against other proteomic datasets. The top three pathways they identified for the biomarker panel were focal adhesion, regulation of the actin cytoskeleton, as well as complement and coagulation cascades. Combining gene expression data with PPI networks and analysis by a computation network method that utilizes PPI affinity has been equally successful in another breast cancer biomarker discovery study. Protein interactors specific for metastatic breast cancer were identified, which unsurprisingly are part of FAC [72]. Like in cancer, FAC has clearly been implicated in the pathogenesis of complex inflammatory diseases including RA [73] and IBD, leading to the tantalizing possibility of clinical biomarkers identified from within the ranks of FAC.

Utilizing single omics technologies with computation biology has provided potential markers, but these have often failed to stand up to rigorous validation due to small sample sizes, differences in tissues sampled, or methodological differences. Perhaps a more holistic, integrated approach is needed to meet the needs of modern medicine. This approach towards a more systemic view necessitates obtaining significant insights by adopting a variety of complementary approaches, such as (1) genomics and transcriptional profiling (including miRNA and lncRNA analysis); and (2) functional and phospho-proteomics (affinity purification and MS), as well as other types of large-scale studies, including lipidomics (isolation and MS analysis of lipid content and protein−lipid interactions), chemical proteomics, and compound screening. With the combined and integrated use of these omics approaches, we can identify potential novel biomarkers and drug targets. All biomarkers to be used in clinical practice need independent validation with clinical samples. One such way as used by Szasz et al. [74] is to merge transcriptomic data from multiple independent datasets to cross validate gene expression biomarkers using univariate and multivariate analyses in 1065 patients. Where such samples are not available or not appropriate, clinical trials with patient cohorts need to be undertaken comparing the biomarker candidates identified against a gold standard. An example of this can be seen in Brandse et al. [75] comparing an inflammatory marker, faecal calprotectin, against the gold standard of leukocyte scintigraphy for denoting inflammatory burden in UC.

## Conclusions

The FAC is a large, dynamic, multimeric structural and signalling opportunity for biomarker identification. Cancer research has led the way with FAC members being implicated as biomarkers of invasion [76], differentiation between normal and cancer cells [77], prognosis [78], and diagnosis [63]. It is clear that the FAC has a role to play in many inflammatory diseases. However, which member, by which mechanism (be it genomic, transcriptomic, proteomic, or a combinatorial approach with a panel of biomarkers [79]) and in which cell type, remains to be formally validated. Here we presented a few examples of how omics approaches could be exploited, separately or in combination, to provide valuable novel biomarkers for inflammatory diseases from members of the FAC that can undergo further validation in a clinical trial.

## Competing interests

The authors certify that they have no conflicts of interest.

## Figures and Tables

**Table 1:** Component examples of the focal adhesion complex

**Category**	**Example**	**Function**	**Refs.**
Actin binding	Actinin1, filamin A, cortactin, zyxin	Crosslink actin; remodel cytoskeleton	[5], [6]

Adapter	SORBS1, ABI1	Link proximal signal pathways; facilitate signal transduction	[7], [8]

Cytoskeletal	Actin, vinculin, plectin, ezrin, paxillin	Facilitate and stabilize signalling platforms; remodel cell shape and movement	[9], [10]

GAP/GEF	DOCK1, ELMO1	Activate small GTPases	[11]

GTPase	Rac1, RhoA	Signal cytoskeletal remodelling, cell growth, phagocytosis, and ruffled borders	[12]

Metalloproteinase	ADAM12	Disintegrin	[13]

PIK/phosphatase	PI3K, INPPL1, PTEN	Regulate AKT/PKB signalling pathway; regulate signalling via IRS proteins	[14], [15]

Receptor	Integrins, IL1R, CD47	Bind to ligands for extracellular matrix constituents including fibronectin and thrombospondin	[16], [17]

Serine/threonine kinase	PAK1, AKT, PRKCA	Effectors linking Rho GTPases to cytoskeletal reorganization; phosphorylate BCL2	[15], [18]

Transcription factor	ITGB3BP	Transcriptional co-regulator	[19]

Tyrosine kinase	FAK, SYK, SRC	Regulate FAC assembly and disassembly	[20], [21], [22]

Tyrosine phosphatase	PTPN1, 2, 6, 11, 12, 22, PTP-PEST	Regulate maturation of focal adhesion; recruit signalling molecules	[23], [24], [25]

*Note*: GTPase, guanosine triphosphatase; GAP, GTPase activating protein; GEF, guanine nucleotide exchange factor; PIK, phosphoinositide kinase; SORBS1, sorbin and SH3 domain-containing 1; ABI1, Abelson interactor 1; DOCK1, dedicator of cytokinesis protein 1; ELMO1, engulfment and cell motility protein 1; Rac1, Ras-related C3 botulinum toxin substrate 1; RhoA, Ras homologue gene family, member A; ADAM12, disintegrin and metalloproteinase domain-containing protein 12; PI3K, phosphatidyl-inositol-3-kinase; INPPL1, inositol polyphosphate phosphatase like 1; PTEN, phosphatase and tensin homologue; IL1R, interleukin-1 receptor type 1; CD47, Cluster of Differentiation 47; PAK1, serine/threonine-protein kinase 1; AKT, RAC-alpha serine/threonine-protein kinase; PRKCA, protein kinase C alpha type; ITGB3BP, integrin subunit beta 3 binding protein; FAC, focal adhesion complex; FAK, focal adhesion kinase; SYK, spleen tyrosine kinase; SRC, proto-oncogene tyrosine-protein kinase; PTPN, tyrosine-protein phosphatase non-receptor type; PTP, protein tyrosine phosphatase; PKB, protein kinase B; Bcl2, B-cell lymphoma 2.
